# Network analysis of time-lapse microscopy recordings

**DOI:** 10.3389/fncir.2014.00111

**Published:** 2014-09-17

**Authors:** Erik Smedler, Seth Malmersjö, Per Uhlén

**Affiliations:** ^1^Unit of Molecular Neurobiology, Department of Medical Biochemistry and Biophysics, Karolinska InstitutetStockholm, Sweden; ^2^Department of Chemical and Systems Biology, School of Medicine, Stanford UniversityStanford, CA, USA

**Keywords:** networks, graph theory, neural networks, cross correlation, small-world, scale-free, calcium signaling

## Abstract

Multicellular organisms rely on intercellular communication to regulate important cellular processes critical to life. To further our understanding of those processes there is a need to scrutinize dynamical signaling events and their functions in both cells and organisms. Here, we report a method and provide MATLAB code that analyzes time-lapse microscopy recordings to identify and characterize network structures within large cell populations, such as interconnected neurons. The approach is demonstrated using intracellular calcium (Ca^2+^) recordings in neural progenitors and cardiac myocytes, but could be applied to a wide variety of biosensors employed in diverse cell types and organisms. In this method, network structures are analyzed by applying cross-correlation signal processing and graph theory to single-cell recordings. The goal of the analysis is to determine if the single cell activity constitutes a network of interconnected cells and to decipher the properties of this network. The method can be applied in many fields of biology in which biosensors are used to monitor signaling events in living cells. Analyzing intercellular communication in cell ensembles can reveal essential network structures that provide important biological insights.

## Introduction

Intercellular communication has been extensively investigated in the adult brain, in which cells form neural network circuits whose activities underlie the basic functions of the brain (Buzsaki, 2004). During the postnatal period, developing networks exhibit spontaneous correlated neuronal activity that plays a central role in their establishment (Katz and Shatz, 1996; Khazipov et al., 2004; Cang et al., 2005; Kandler and Gillespie, 2005; Nicol et al., 2007). In developing neocortical structures, synchronized activity has been observed at scales ranging from correlated pairs of neural progenitor cells (Owens and Kriegstein, 1998) to gap junction-synchronized cortical columns (Yuste et al., 1992; Kandler and Katz, 1998; Dupont et al., 2006). To understand the nature and physiological roles of these networks, it is essential to identify them and characterize their structures.

**Figure 1:**
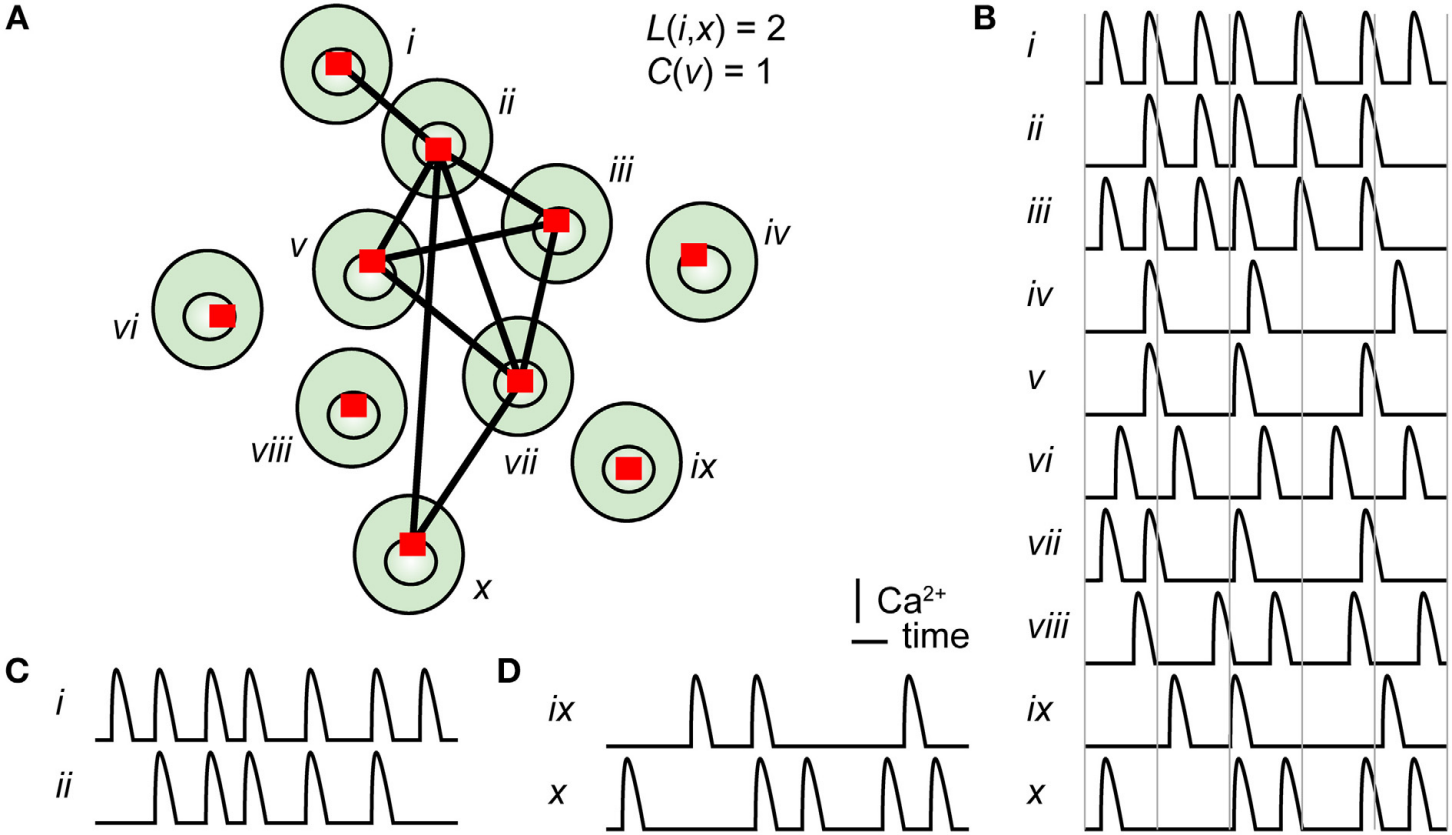
**Principle of cross-correlation analysis of a cell signaling network. (A)** Illustration of a group of ten cells, numbered with roman numerals, with links connecting strongly correlated cells. The shortest path length between cell *i* and cell *x*, as well as the clustering coefficient for cell *v*, is stated in the figure. The shortest path between cell *i* and cell *x* is 2. Cell *v* has three connected neighbors, and they are all neighbors of each other; thus, the clustering coefficient is 3/3 = 1. **(B)** Pseudo Ca^2+^ concentration vs. time traces for all ten cells. Two time series with high **(C)** or low **(D)** correlation coefficients.

Graph theory is the study of graphs (mathematical term for networks) consisting of nodes (also called vertices) and links connecting the nodes (also called edges) (Feldt et al., 2011). Many relations and dynamic processes can be modeled by graphs, in such diverse fields as biology, social sciences, information systems, and transportation systems (Newman, 2010). When studying biological networks, a typical node can represent a cell (Malmersjö et al., 2013a), specific brain area (Biswal et al., 2010), or protein (Jeong et al., 2001). A link between two nodes can represent a functional interaction, e.g., via ion fluxes (Malmersjö et al., 2013a), synapses (Bonifazi et al., 2009), physical connections by axon bundles (Sporns et al., 2005), or protein–protein interaction (Jeong et al., 2001). The method presented here uses cross-correlation analysis and graph theory to evaluate network topologies among living cells (Figure 1A) exhibiting spontaneous Ca^2+^ signaling (Figure 1B). The analysis identifies cell pairs with highly correlated Ca^2+^ signals (Figure 1C) or with uncorrelated Ca^2+^ signals (Figure 1D). Furthermore, the method can also reveal highly connected “hub cells” (scale-freeness) and high clustering accompanied by short internodal distances (small-worldness). Such functional network designs are effective in many types of biological systems (Barkai and Leibler, 1997; Barabasi and Oltvai, 2004). Furthermore, network analysis has also been used in fMRI based studies of cognitive neuroscience (Sporns, 2014). By comparing the activity of healthy and diseased subjects, changes in network structure and activity can be linked to physiology and pathophysiology (Honey and Sporns, 2008).

We developed a method to identify and characterize network structures from time-lapse microscopy recordings, and implemented it in MATLAB. The resultant software tool is easy to extend and use. The method was originally designed to study network formations among large populations of neural progenitor cells exhibiting spontaneous intercellular Ca^2+^ activity (Malmersjö et al., 2013a,b). However, this method could potentially be used to analyze any kind of cellular activity that is detectable in living single cells by time-lapse microscopy, e.g., contractile activity measured by bright-field microscopy (Kitambi et al., 2012) or activity measured using various biosensors, such as NF-κ B, cAMP, or ERK (Nelson et al., 2004; Malmersjö et al., 2010; Aoki et al., 2013; Everett and Cooper, 2013). Thus, this method could be used to identify and investigate the details of uncharacterized network structures.

## Materials and methods

### Calcium imaging

Cells were loaded with the Ca^2+^-sensitive fluorescence indicator Fluo-3/AM (5 μM, Invitrogen) at 37°C for 30 min in Krebs-Ringer's buffer containing 119.0 mM NaCl, 2.5 mM KCl, 2.5 mM CaCl_2_, 1.3 mM MgCl_2_, 1.0 mM NaH_2_PO_4_, 20.0 mM HEPES (pH 7.4), and 11.0 mM dextrose. Measurements of cytosolic Ca^2+^ were carried out in Krebs-Ringer's buffer at 37°C using a heat-controlled chamber (QE-1; Warner Instruments) and a cooled electron-multiplying charged-coupled camera (QuantEM 512SC, Photometrics) mounted on an upright fixed stage microscope (Axio Examiner.A1, Carl Zeiss) equipped with a 20× 1.0 N.A. lens (Carl Zeiss). Excitation at 480 nm was assessed with an illumination system (DG4, Sutter Instrument) at sampling frequency of 0.2–1 Hz (*T* = 1–5 s). MetaFluor (Molecular Devices) was used to control all devices and to analyze acquired images. The cell-free area was created by making a cut with a fine syringe (BD Microlance™ 3, 0.4 × 19 mm) in confluent HL-1 cells. Dishes were placed in an incubator for 5 h before imaging.

### Cell culture

Neural progenitors were derived from mouse embryonic stem cells as described before (Malmersjö et al., 2013a). HL-1 cells were cultured as previously described (Claycomb et al., 1998).

### Cross-correlation analysis

Cross-correlation was used to determine whether two cells were functionally interconnected. Cross-correlation analysis is a mathematical method for quantifying the linear similarity between two waves as one of them is shifted in time (Brockwell and Davis, 1998). When cross-correlation analysis is applied in signal processing, the waves are typically time series consisting of discrete sets of data points [*X*_*t*_, *t* ∈ *T*], e.g., images acquired by time-lapse microscopy. The normalized version of the cross-correlation function, i.e., the cross-covariance, is commonly used for image-processing applications in which the brightness of the image is the quantitative measure. In MATLAB, the cross-covariance is implemented as *xcov*:

(1) $$c_{xy}\left(m\right)=\{\begin{array}{l} \begin{array}{l} \sum_{n\;=\;0}^{N\;-\;\left|m\right|\;-1}\left(x\left(n+m\right)-\frac{1}{N}\sum_{i\;=\;0}^{N\;-\;1}x_{i}\right)\\ \;(y_{n}^{*}-\frac{1}{N}\sum_{i\;=\;0}^{N\;-\;1}y_{n}^{*})\;if\;m\geq 0\\ c_{yx(-m)}^{*}\;if\;m<0 \end{array} \end{array}$$

Here, *m* is the lag, *N* is the number of time points, *n* is the summation index, and *x* and *y* are the two time series. Because *N* is a finite number, the above function (Equation 1) is just an estimation of the real cross-covariance function:

(2) $$\varphi_{xy}\left(\mu \right)=E\left[\left(x_{n\;+\;m}-\mu_{x}\right)\left(y_{n}-\mu_{y}\right)\right]$$

Here, μ_*x*_ and μ_*y*_ are the mean values of the stochastic processes (the time series are modeled as stochastic) and *E* is the expectation value operator (the average value from multiple samples). If time is fixed in the cross-covariance function (Equation 2), it will result in the well-known correlation coefficient, also known as the Pearson correlation, a real number between -1 and 1. A correlation coefficient equal to 0 indicates no linear relation between the waves, whereas a coefficient equal to 1 or -1 demonstrates a perfect linear relation.

**Figure 2:**
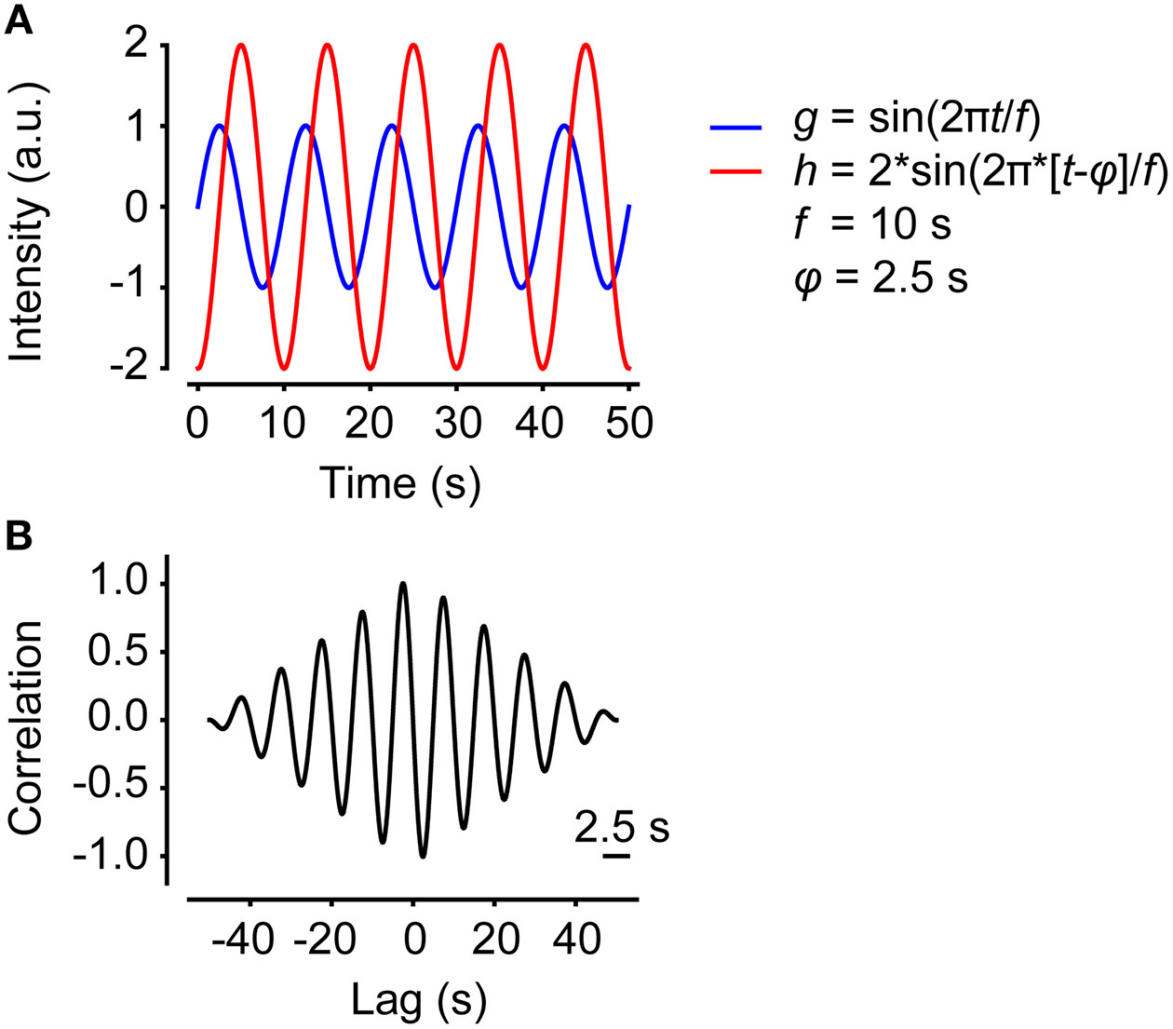
**Correlation as a function of lag. (A)** Two sine functions with the same frequency but different amplitudes and phases, plotted in the same graph. **(B)** The correlation as a function of lag of the two sine waves in **(A)**.

Two time series might be highly correlated even if one of them is shifted in time. Calculating the correlation as a function of lag enables determination of the maximum correlation despite lag. Figure 2A shows two sine waves with identical frequency, but different amplitudes and phases. Figure 2B shows correlation as a function of lag for the two sine waves. The phase shift is 2.5 s. Note that the correlation function is amplitude-independent and only considers the relative amplitude. In some cases, for example neurons interconnected with synapses, the identified lag could be related to the pausing time between two neurons. However, most often this effect is interpreted as an effective phase shift.

Before calculating the correlation between two signals, they can be filtered by subtracting underlying trends; this process is called trend correction. For instance, bleaching or focus shifts might lead to a gradual decay, superimposed on the actual signal. By fitting the signals to a polynomial function with a certain degree (for example a linear function for linear trends), this effect can be reduced.

**Figure 3:**
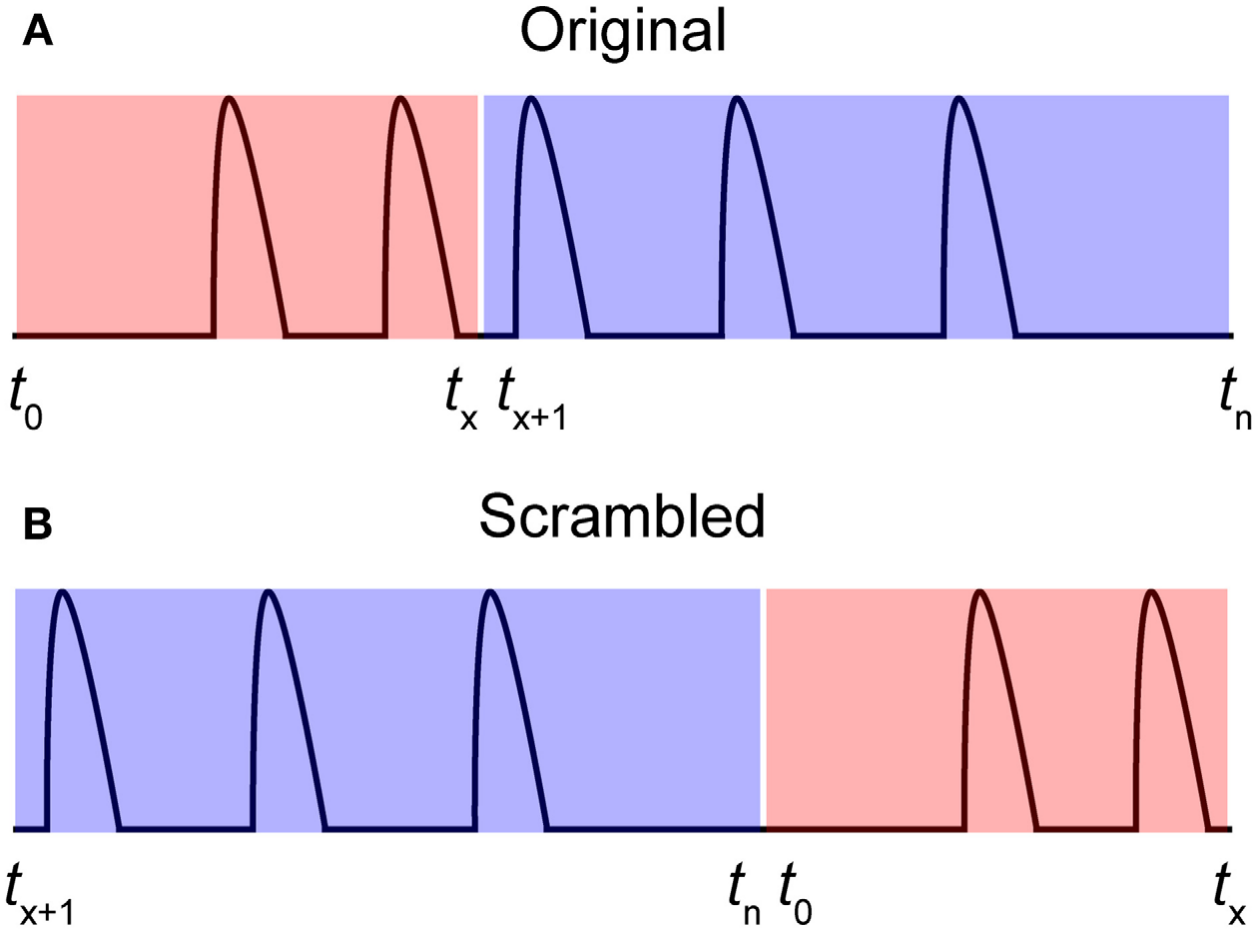
**Producing a scrambled signal. (A)** A pseudo-signal with start and end time points *t*_0_ and *t_n_*. **(B)** A scrambled signal was created by shuffling the signal in **(A)** to a random time point *t*_*x*_.

It is important to decide a cut-off that filters out insignificant correlations. We have developed a method for determining such cut-off values using a scrambled data set. A scrambled data set *f*_*scrambled*_ is created by shuffling the individual time series *f* to random starting points *t* (Equation 3). Thus, each original time series is divided into two parts at a random position and then put together again in the opposite order. Figure 3 illustrates a time series between *t*_0_ and *t*_*n*_ (Figure 3A) that is shuffled to *t*_*x*_ (Figure 3B). This procedure is then repeated for all-time series in the data set in a cyclic fashion:

(3) $$f_{scrambled}\left[1:N\right]=(f\left[t:N\right]\;f\left[1:t-1\right])$$

The total activity in the original data set and the scrambled data set are thereby conserved. The mean or the 99th percentile of the cross-covariance values of the scrambled data set can then be applied as the cut-off value.

### Graph theory

**Figure 4:**
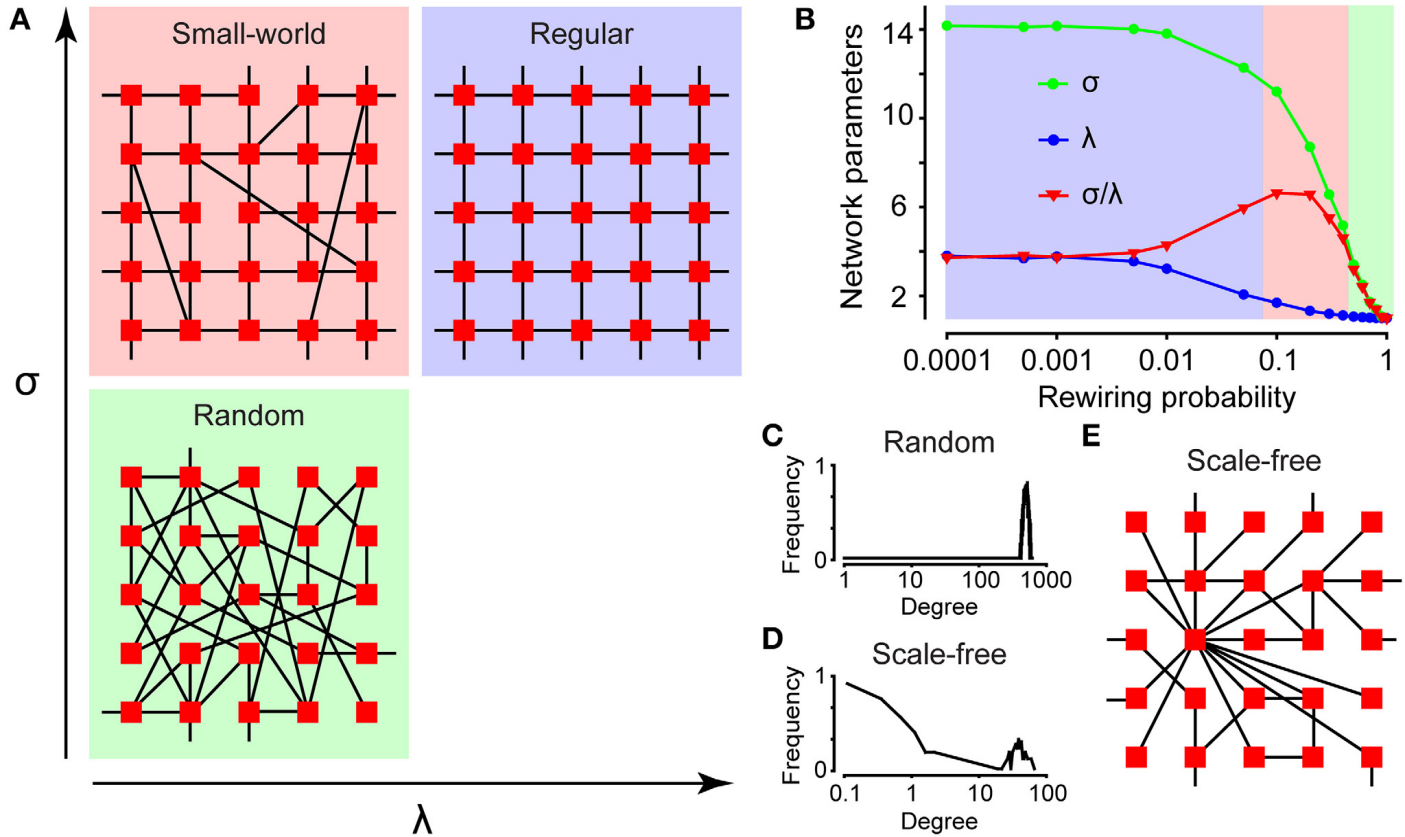
**Characterizing network structures. (A)** The topologies of a small-world (red), regular (blue), and random (green) network depicted with their internal relations to the network parameters σ and λ. **(B)** The network parameters σ and λ for a small-world (red), regular (blue), and random (green) network, plotted as a function of rewiring. The degree distributions for a random **(C)** and scale-free **(D)** network plotted in a log–log scale. **(E)** The topology for a scale-free network.

Graph theory can be used to characterize the topology of a network (Feldt et al., 2011). In graph theory, several network parameters are calculated for each network to determine the network's type (Newman, 2010), shown in Figures 4A,B. Connectivity is here defined as the number of cells with a correlation coefficient larger than the cut-off, divided by the total number of cells. This is a measure of the degree of connections for the network, because a high cut-off results in a low connectivity and vice versa. The edge density, also referred to as connectance (Newman, 2010), is defined as the number of edges divided by the maximum number of edges. The neighbors of a node are all the nodes connected to it in one step. The degree of a node is its number of neighbors; hence the degree distribution *P*(*k*) of a network is the distribution of nodes with a degree equal to *k*. *P*(*k*) is obtained by counting the number of nodes *N*(*k*) with *k* = 1, 2, 3, … connections and dividing by the total number of nodes.

Classical models of networks occurring in nature are either regular or random, as shown in Figure 4A. In a regular network, each node is connected to *k* other nodes (Feldt et al., 2011). Figure 4A shows a regular network with *k* = 4. In a random network, nodes are connected with links stochastically, resulting in a Poisson-shaped degree distribution around *pN*, where *p* is the probability and *N* is the total number of nodes, plotted in panel Figure 4C. In this example, 1000 nodes are randomly connected to each other with 50% probability.

Small-world networks combine features of both regular and random networks, with high clustering as in regular networks but short internodal distances as in random networks (Watts and Strogatz, 1998). According to the Watts-Strogatz model, a small-world network can be generated by randomly rewiring links in a regular network (Watts and Strogatz, 1998). The properties of small-world networks are assessed by calculating the mean clustering coefficient *C* and the mean shortest path length *L* of the network using the MatlabBGL library (http://www.mathworks.se/matlabcentral/fileexchange/10922-matlabbgl). Hence, a small-world network is characterized by the following relations:

(4) $$\sigma =\frac{C}{C_{rand}}\gg 1\;and\;\lambda =\frac{L}{L_{rand}}\approx 1$$

The clustering coefficient is the number of neighbors of a node that are also neighbors of each other divided by the total number of possible links between the neighbors, as shown in Figure 1A. Thus, it reflects the number of groups in a network. The shortest path length is the minimum number of nodes that must be passed to travel from one node to another, as depicted in Figure 1A. The values of *C* and *L* are then compared with the corresponding values of *C_rand_* and *L_rand_* for a randomized version of the network. Figure 4A shows a simplified scheme for classification of small-world (red), regular (blue), and random (green) networks according to their mean clustering coefficient σ and mean shortest path length λ. The interrelationship between the clustering coefficient (σ), shortest path length (λ), and small-world parameter (σ/λ) as a function of rewiring probability of a regular network is shown in Figure 4B. In this simulation, 1000 nodes were connected in a circle to their two closest neighbors, and random links were rewired with increasing probability.

A network is defined as possessing small-world characteristics if the mean path length is as short as in the corresponding random network, whereas the mean clustering coefficient is higher. At the level of the central nervous system, small-world networks are thought to promote efficient information flow at a low wiring cost (Achard and Bullmore, 2007).

The Barabási–Albert model of preferential attachment states that a scale-free network can be generated by allowing a random network to grow according to preferential attachment (Barabasi and Albert, 1999). If the degree distribution approximately follows a power law (a heavy-tailed function without a clear mean value or scale), the network is defined as scale-free.

(5) $$P\left(k\right)\propto k^{-\gamma }↔\log\left(P\left(k\right)\right)\propto -\gamma \log(k)$$

In Figure 4D the degree distribution is plotted in a log–log scale that shows a linear function if possessing scale-free characteristics. The scale factor γ is inferred using the least-squares method; however, some authors have argued that the maximum likelihood method is superior (Clauset et al., 2009). Scale-free networks have some nodes with many neighbors that can act as hubs (Barabasi and Albert, 1999). On theoretical grounds, these networks are thought to be robust (Albert et al., 2000): as depicted in Figure 4E. In Figure 4D, a network with 100 start nodes with random connections was grown by connecting 900 new nodes in a “rich-get-richer” fashion.

### Software

MATLAB version 7.12.0 (http://www.mathworks.com), or later, including the Signal Processing toolbox, is required to implement the analytical method we describe here. The current versions of MATLAB run on multiple operating systems. NetworkIdentification.m and NetworkAnalysis.m are the files containing the main code, supported by the function files mic2net.m, crosscorrelation.m and pickcells.m. The program is initiated by mic2net.m. All files are included as supplementary material for this article (Supplementary Materials). The MatlabBGL library for MATLAB is required for the topology analysis that calculates the shortest path length and clustering coefficients, and is provided as supplementary material online. The original source for the MatlabBGL can be found elsewhere: (http://www.mathworks.com/matlabcentral/fileexchange/10922).

Fiji (http://www.fiji.sc/Fiji) or any other image-processing software that can extract the mean intensities, x-coordinates, and y-coordinates from multiple regions of interest (ROIs) in time-lapse microscopy recordings is required to analyze time-lapse microscopy recordings.

## Results

**Figure 5:**
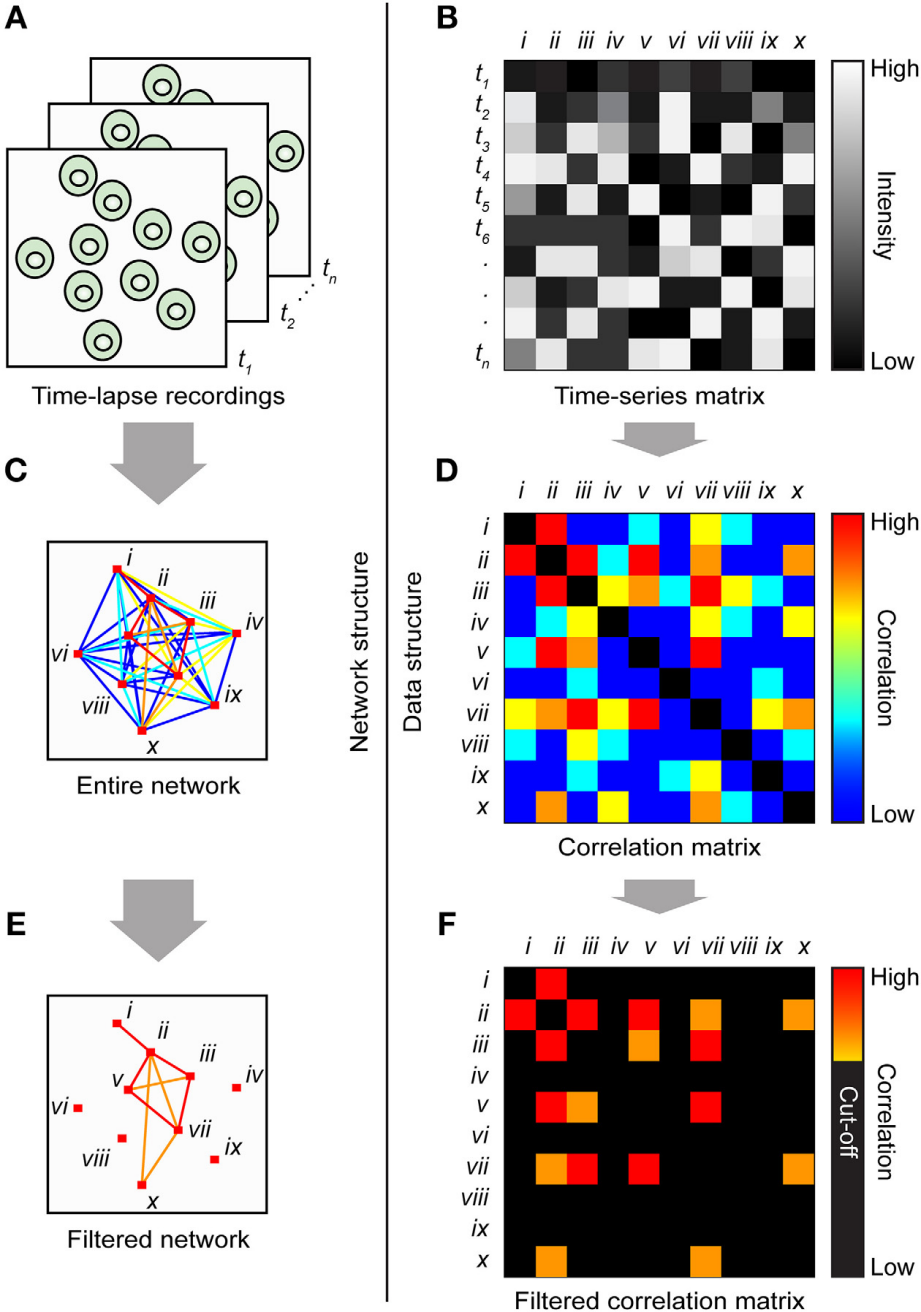
**Cartoon illustrating the basic steps of the method. (A)** A time-lapse cell recording is imported into image-processing software that calculates the mean intensity and x,y-coordinates for each ROI and saves all the values in a tab-delimited text file. Time points are indicated by *t_1_*, *t_2_*, … *t_n_*. **(B)** The file is loaded into MATLAB, which generates a matrix in which the individual cells' time series are divided into columns and the time points are divided into rows. The ten cells are indicated by roman numerals. The entire network structure is plotted **(C)** from the correlation matrix **(D)**. Colored lines and matrix elements indicate the strength of correlation between cell pairs. Significant correlations were plotted **(E)** by applying a cut-off to the correlation matrix **(F)**.

The first step of the analysis is to extract data from the time-lapse microscopy recordings using a fluorescent biosensor to monitor e.g., intracellular Ca^2+^ signaling (Figures 5A,B). This is carried out by calculating the mean fluorescence value of each cell marked with an ROI. Because network structures are spatial structures, we also need the x- and y-coordinates of each cell. Each ROI is therefore assigned its x- and y-coordinates. The values are saved in a file to be loaded into MATLAB. Next, all possible combinations of cell pairs are examined using cross-correlation analysis (Figures 5C,D). A correlation value between 0 and 1 is calculated for each cell pair that reflects the strength of interaction. Note that only the absolute value of the cross-correlation function is saved in the correlation matrix, thus neglecting the difference between correlation and anti-correlation. To discriminate between real and false correlations, a cut-off value should be established (Figures 5E,F). Our method provides an unbiased way to calculate such cut-off values using signals that have been scrambled by randomly shuffling each cell signal in the time domain (see Section Materials and Methods); thus the total activity within the data set is preserved. Thereafter, the cut-off value can be set as the 99th percentile of all correlation coefficients within the scrambled data set (Malmersjö et al., 2013a,b). Thus, two cells are defined as interconnected if their correlation coefficient exceeds the cut-off value.

**Figure 6:**
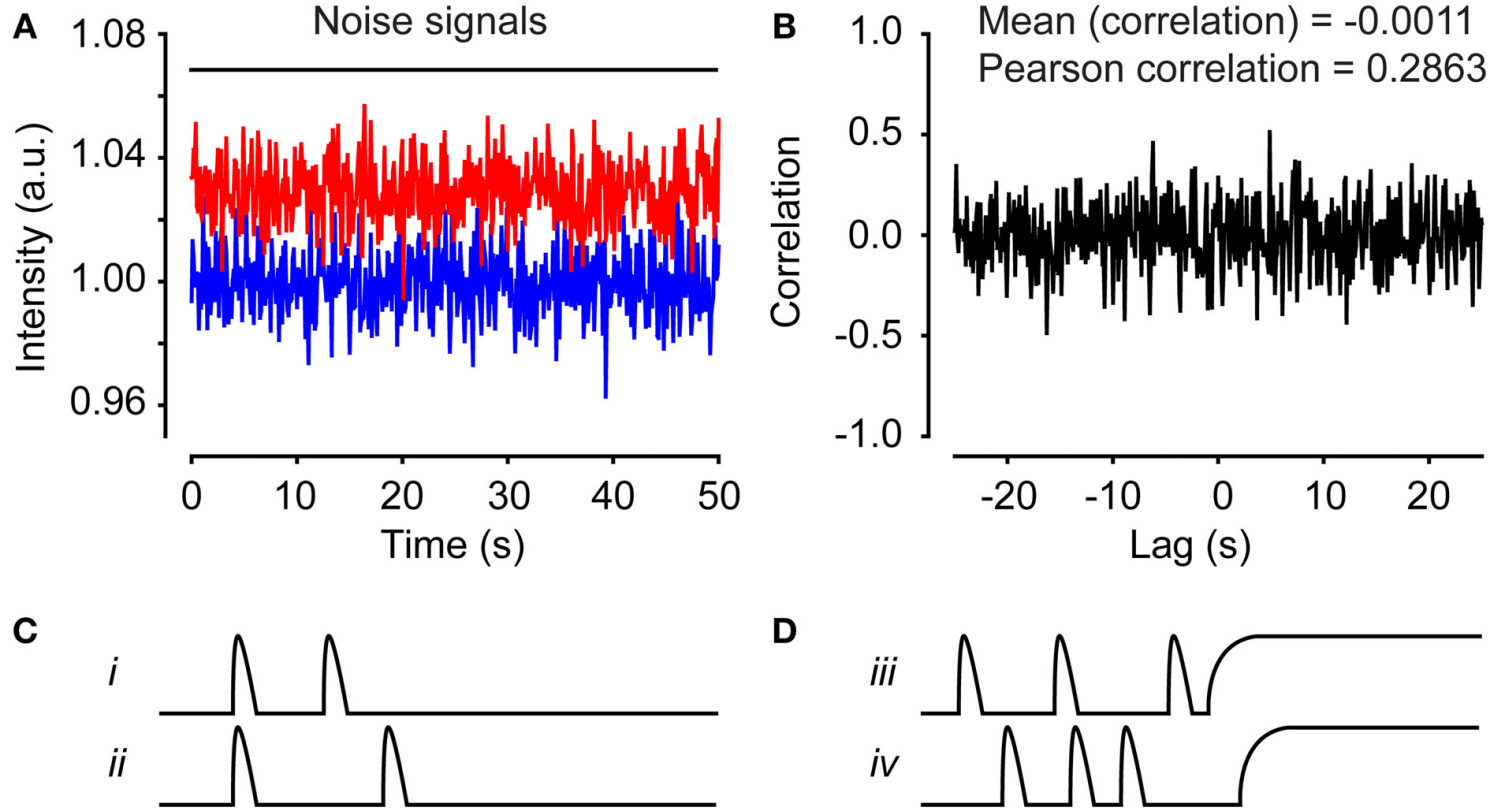
**Time series can produce false correlations. (A)** Two separate time series consisting of pseudorandom numbers normally distributed. **(B)** The cross-correlation calculation for the time series in **(A)**. The mean correlation is −0.0011 and the Pearson correlation is 0.2863. Illustration of false-positive cell pairs with high correlations due to small numbers of peaks **(C)** or sustained plateau increases **(D)**.

We applied this method to the investigation of functional networks in neural progenitor cells exhibiting spontaneous Ca^2+^ activity. Before imaging, neural progenitor cells derived from embryonic stem cells were loaded with the Ca^2+^ indicator Fluo-3/AM for 30 min. Time-lapse recording was performed by fluorescence microscopy, using a sampling frequency of 0.2 Hz. After approximately 30 min of recording, the time-lapse experiment was terminated, and the network analysis could start. Using the Fiji image-processing software, each cell was marked with an ROI and the mean fluorescence value was calculated (see Supplementary Movie 1 for a step-by-step guide). The values were saved in a tab-delimited text file that can be loaded into MATLAB (see Supplementary Movie 2 for a step-by-step guide). The sampling frequency and the physical pixel size were stated explicitly in MATLAB. Signal artifacts, caused, e.g., by focus change and/or drug application, were filtered out by choosing a “clean” time window for the analysis. Because a data set containing cells with no activity will result in a correlation analysis with false positives, only active cells were chosen for further analysis (Figures 6A,B). Cells with very few peaks (Figure 6C) or dying cells that create a sustained plateau increase in the signal (Figure 6D) can also result in false positives. Active cells could be defined, for example, as cells exhibiting three transient intensity increases or more that exceed 15% over the baseline (Malmersjö et al., 2013a) or more than two standard deviations over the noise. Here noise is defined as the baseline fluctuations of a non-active cell. If the data set has a low signal-to-noise ratio, digitization is required. The process of digitization replaces numbers that do not exceed the threshold with 0 and all others with 1. To further optimize the data set before analysis, single-cell signals can be trend-corrected and/or maximal lag can be selected (see Section Materials and Methods). Thereafter, it is advisable to manually check cell pairs with high correlation coefficients in order to filter out false positives. Another source of false positive correlations could arise from defining two separate ROIs that inadvertently overlaps covering parts of the same cell. To avoid this, an alternative approach would be to automatically segment cells with a nuclear marker used as a mask.

**Figure 7:**
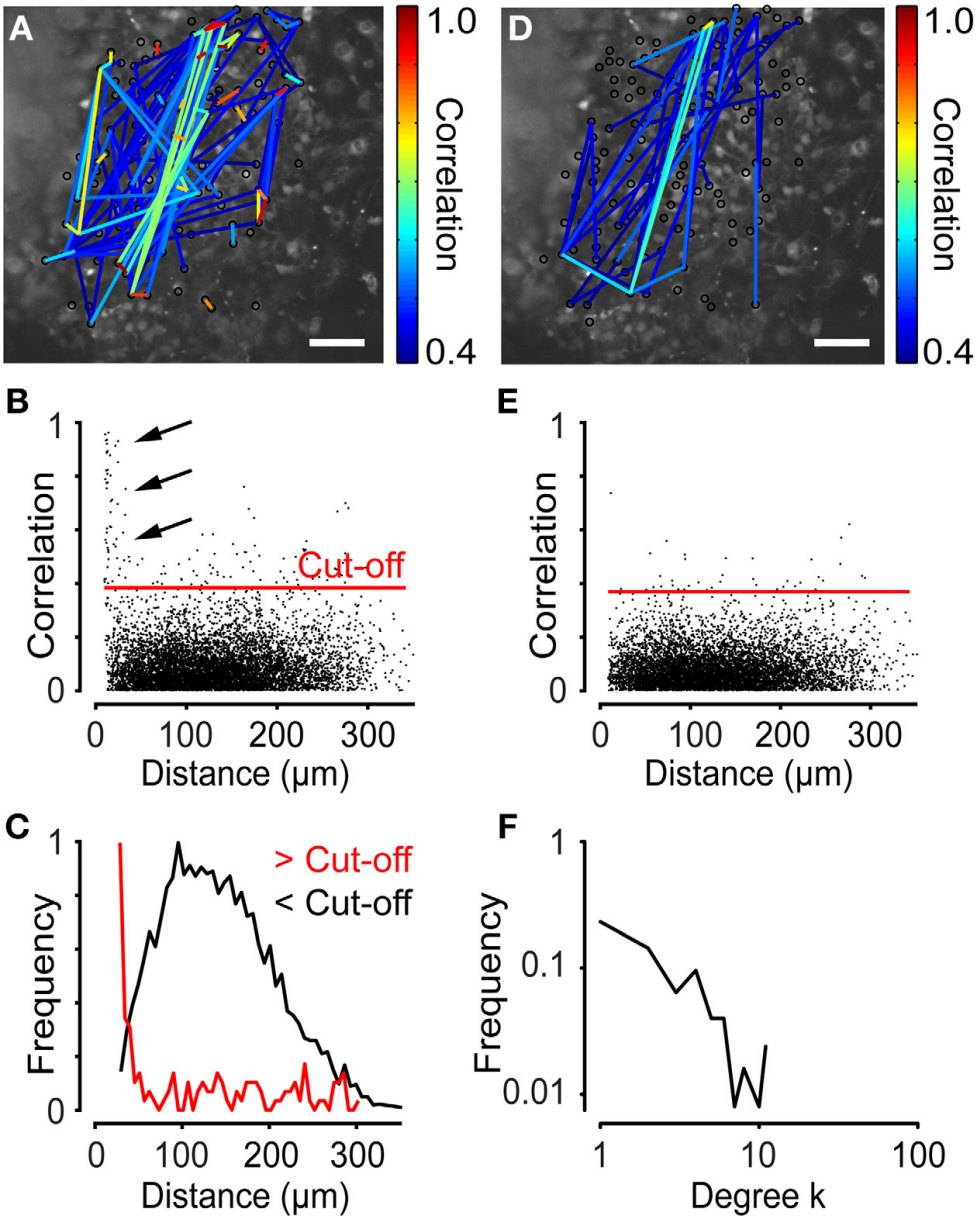
**Network analyses performed on time-lapse Ca^2+^ recordings of neural progenitor cells. (A)** Network with a cut-off of 0.39 for neural progenitor cells plotted on top of a microscopic image. **(B)** Correlation as a function of distance for the experiment shown in **(A)**. Red line indicates the cut-off level. Arrows indicate cell pairs with high correlations and short intermodal distances. **(C)** Distance distribution of data in **(B)** with correlations above cut-off (>Cut-off), shown in red, and below cut-off (<Cut-off), shown in black. Note the higher frequency of shorter distances for correlations above the cut-off. **(D)** Network plot of data from **(A)** scrambled in time domain, as described in Figure 3. **(E)** Correlation as a function of distance for the data shown in **(D)**. **(F)** Degree distribution of the analysis on neural progenitor cells showed in **(A)**. Scale bars, 50 μm.

The network analysis generates a network structure that can be plotted on top of a microscope image. Figure 7A shows the network plot of correlation coefficients for neural progenitor cells exhibiting spontaneous Ca^2+^ activity. Only connections exceeding a cut-off (see below) were plotted. The network plot suggests that neighboring cells are strongly correlated (Figure 7A). By studying the distance distribution of the correlation coefficients in detail, we indeed observed that cells close to each other were strongly correlated (Figures 7B,C). We previously tested this observation experimentally by pharmacological inhibition of gap junctions with Octanol (1 mM) and shRNA knockdown of the *Connexin 43* gene, both of which disrupt the network activity (Malmersjö et al., 2013a). As described above, the cut-off was determined by calculating the mean of the 99th percentile of the scrambled data (see Section Materials and Methods). Plotting the distance distribution of the scrambled data revealed no cell pairs with high correlations and short internodal distances (Figures 7D,E). Thus, the cut-off was set to 0.39 to filter out the bulk of cells with low correlation values (Figures 7B,C,E; red lines). The degree distribution revealed a typical scale-free network structure (Figure 7F). Calculation of the network parameters σ = 7.7 ± 0.92, λ = 0.97 ± 0.056, and γ = −1.2 ± 0.049 (*N* = 6) yielded a characterization of this network as a small-world network with scale-free topology.

**Figure 8:**
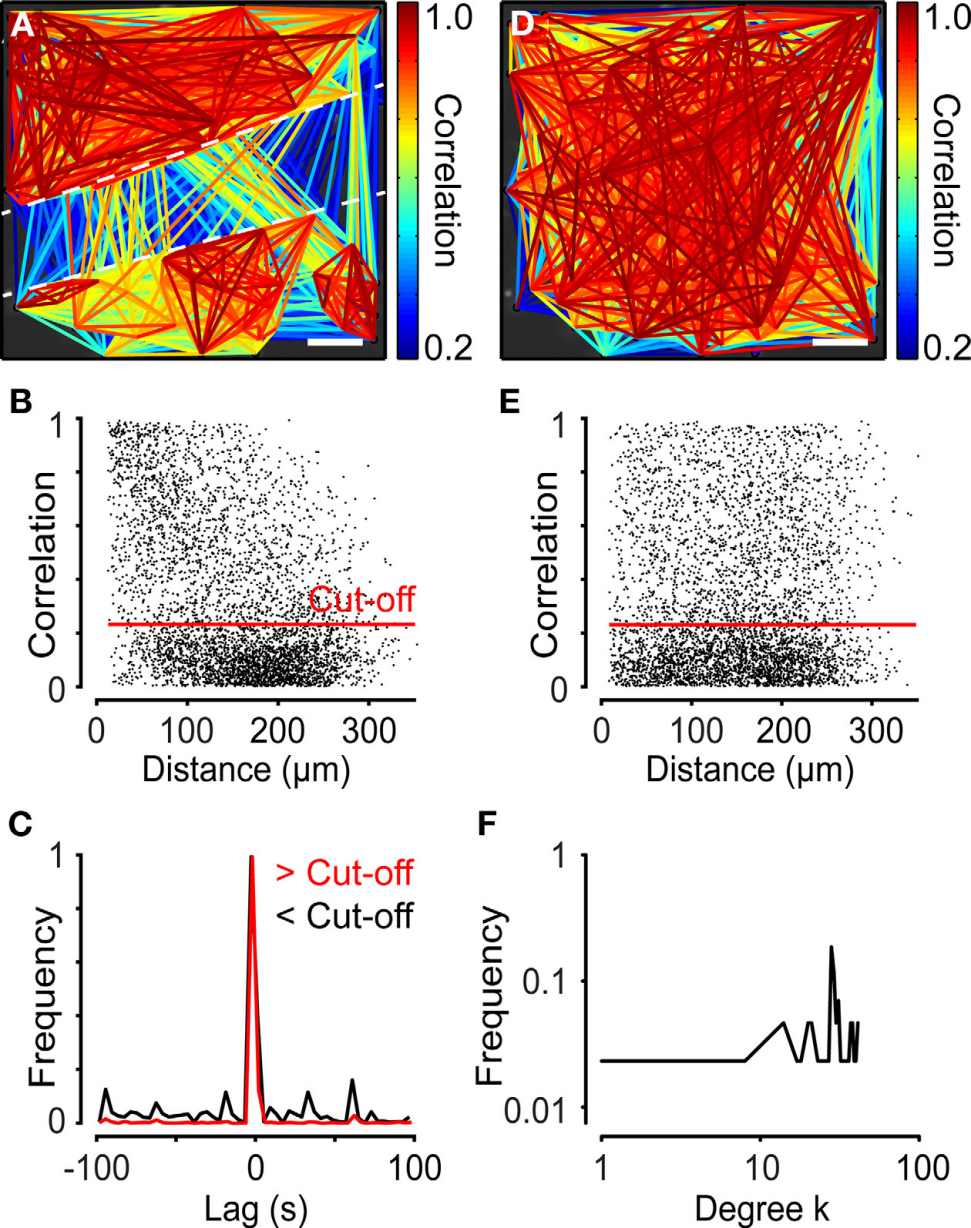
**Network analyses performed on two separated cell ensembles in cardiac HL-1 cells. (A)** Network of atrial HL-1 cells with a cut-off of 0.23, plotted on top of a microscope image. A cut divided the cells into two populations, as indicated in the figure. **(B)** Correlation as a function of distance for the experiment shown in **(A)**. **(C)** Lag distribution for the maximum correlations above cut-off (>Cut-off), shown in red, and below cut-off (<Cut-off), shown in black. Note the small peaks for values below cut-off. **(D)** Network plot of data from **(A)** with x-y-coordinates of all cells randomized. **(E)** Correlation as a function of distance for the data shown in **(D)**. **(F)** Degree distribution of the analysis of the HL-1 cells shown in **(A)**. Scale bars, 50 μm.

Network analysis was also performed on cardiac HL-1 cells interconnected by gap junctions (Claycomb et al., 1998). These cells exhibited spontaneous Ca^2+^ activity with network properties (Figure 8A). The analysis revealed that these networks did not have small-world characteristics (σ = 1.06 ± 0.020 and λ = 1.0 ± 0.0083, *N* = 4). Instead, the HL-1 networks showed similarities to a random network. To test the strength of the method, the network was divided into two sub-networks by creating a cut with a syringe (Figure 8A). The distance distribution of HL-1 cells revealed a majority of cell pairs with high correlations and short internodal distances (Figure 8B), although not as clearly as in the neural progenitor cells described above (Figure 7B). Studying the maximal correlation as a function of lag revealed a highly synchronized cell population (Figure 8C). Next, we tested the dependency of correlations on distance by randomizing the cell positions of the cells. After randomizing the cells' coordinates, the cut was no longer visible (Figure 8D). Plotting the distance distribution of the data with randomized positions resulted in more evenly spread cell pairs (Figure 8E), revealing that the randomization abolished the dependency of correlation on distance (Figures 8D,E). In this analysis, the cut-off was set to 0.23, using the same method as described above for neural progenitor cells, to filter out the bulk of cells with low correlation values. Overall, the HL-1 network exhibited similarities with a random network, as indicated by the degree distribution (Figure 8F).

Usage of our method will generate a number of different parameter values describing the corresponding functional network: mean value of all correlations, mean value of correlations above cut-off, 99th percentile of all correlations, connectivity, edge-density, λ, σ, small-world parameter σ/λ, and γ. How to statistically compare and evaluate networks is non-trivial and still being studied. As a first approximation, different networks of different type of cells or treatments can be statistically compared using for instance the Student's *t*-test, thus assuming normally distributed values (Malmersjö et al., 2013a). If, for example, one treatment is hypothesized to decrease the overall connectivity within a network, mean values of a number of samples of edge-density and connectivity can be calculated and compared between groups. However, it is more accurate to compare several parameters simultaneously and perform a Bonferroni correction to counteract the problem with multiple comparisons. Using a Bonferroni correction sets a lower bound on the significance threshold. Regarding degree distributions, for example the scale factor for power-law distributions can be inferred using standard least-square or the maximum likelihood method (Clauset et al., 2009).

## Discussion

The method described here identifies network formations in data sets acquired by live-cell imaging and enables objective analyses of these networks by characterizing their network topology. The information provided here, together with the accompanying software tools, should enable the experimental biologists without programming skills to identify and quantitatively analyze networks of time-lapse microscopy recordings. The crucial steps of pre-processing the data and determining the cut-off are discussed further below. Comparisons with other network methods and similarities between cell networks and social networks are also discussed.

### Data pre-processing

The outcome of the analysis depends critically on the quality of the input data. It is therefore essential to pre-process the data; for example, by selecting a time window that includes no artifacts or performing trend correction to remove the influences of fluorescence/dye bleaching or dye leakage. It is also crucial to pre-select cells that are active: the cross-covariance function only considers the relative amplitude; consequently, inactive cells with normal background noise appear correlated in the program, resulting in false-positive network links (Figures 6A–C). Therefore, inactive cells should be manually removed from the data set. Cells with sustained plateau increases may also result in false-positive network links (Figure 6D).

### Determining the cut-off

**Figure 9:**
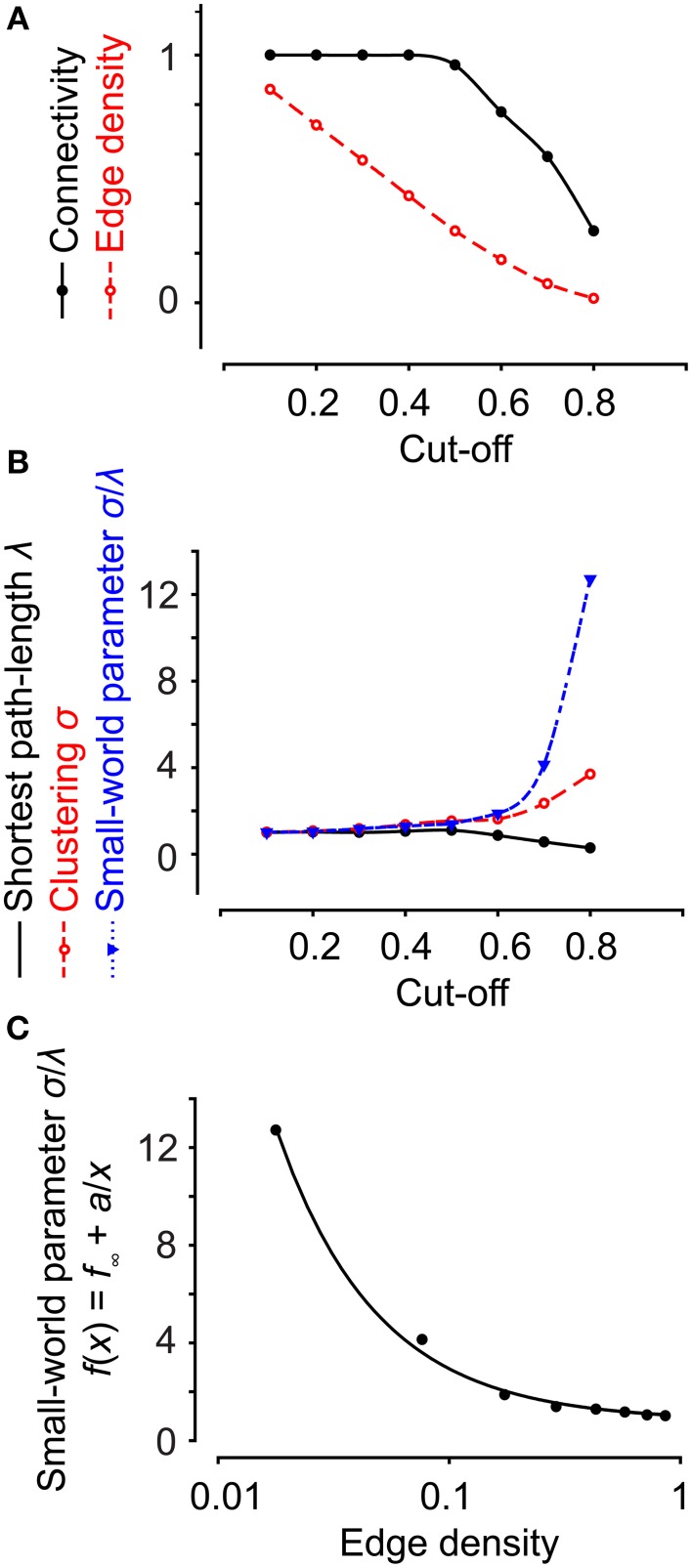
**Network properties are dependent on the cut-off value. (A)** Increasing the cut-off for an experiment on neural progenitor cells decreased both the edge density and connectivity. **(B)** Increasing the cut-off decreased the shortest path length and increased clustering and the small-world parameter. **(C)** The small-world parameter does not exhibit a linear dependency on the edge density. Data were fit to a polynomial function of the first order inverse.

In the two experiments above, different cut-off values were used to filter out only strongly connected cells. The cut-offs were chosen as the mean of the 99th percentile of the correlation coefficients for the scrambled data. Changing the cut-off affected the edge density and the connectivity, two different definitions of the number of connected cells (Figure 9A). In addition, the small-worldness of a network is strongly related to its edge density (Humphries and Gurney, 2008). As illustrated by a functional network of neural progenitors, increasing the cut-off (the same as decreasing the edge density) increases the small-world parameter (Figures 9B,C). Thus, it is very important to choose an adequate cut-off; for example, by studying the correlation distribution of a scrambled data set. It is worth pointing out that a network of strongly correlated cells does not automatically have the properties of small-worldness or scale-freeness, as is clear from a theoretical point of view (Watts and Strogatz, 1998; Barabasi and Albert, 1999) and from our two examples of highly correlated cells with different network structures (Figures 7A, 8A).

### Comparison with previously published products

Our method allows identification and analysis of network structures from cell-imaging data sets acquired by time-lapse microscopy using code written in MATLAB. MATLAB is a widely used numerical computing environment and programming language developed by MathWorks (http://www.mathworks.com). A number of software tools for analyzing networks exist, but these are most often suited for genetic and molecular data, rather than time-lapse microscopy recordings (Brohee et al., 2008; Doncheva et al., 2012). LEDA (Mehlhorn and Naher, 1995) (C++) and QuACN (Mueller et al., 2011) (R) are tools for users with programming skills, whereas Pajek (Batagelj and Mrvar, 2004), Cytoscape, and yED are more graphically oriented solutions. A common feature of all of these software tools is that they are primarily used to analyze already identified networks, and are therefore not applicable to data sets consisting of time-lapse recordings of unknown network activity.

### Cell networks vs. social networks and transportation systems

We hypothesized that the scale-free network structure exhibited by neural progenitor cells (Figure 7A) was a consequence of the fact that dividing cells are more prone to connect to already existing highly connected nodes, in accordance with the Barabási–Albert model of preferential attachment (Barabasi and Albert, 1999). Highly active neural progenitors with many neighbors divide more frequently and tend to connect to their daughter cells. The same phenomenon exists in social networks, in which people with many friends can acquire new friends more easily than those with few. By analogy to the Watts-Strogatz model of long shortcuts (Watts and Strogatz, 1998), the small-worldness in the central nervous system could be generated by extensions from neurons that connect to other neurons at relatively long distances, thereby decreasing the mean internodal distance. Examples of small-world networks include social networks of actors in Hollywood, in which the distance between two random actors is shorter than expected and cliques are present (Watts and Strogatz, 1998). An example of a scale-free small-world network is how airlines connect the world through nodes of airports (Guimera et al., 2005). A random disruption to one of the thousands of airport around the world would usually not disturb the flow of travelers, but a shutdown of a hub, such as London Heathrow Airport, could severely harm the network. Hence a scale-free small-world network has a good tolerance for random deletion of nodes, but low tolerance for a directed attack to a hub. Graph theory predicts that such network designs are effective for biological systems, since they enable efficient information transfer and robustness against failure of single cells (Barabasi and Oltvai, 2004).

### Future directions

Studying network structures is becoming increasingly popular in many subfields of biology, in part because network designs of biological systems resemble the Internet, social networks, and transportation systems. Studies of networking in cell biology and social networks will benefit from each other and further our knowledge of how cells interact to build a functioning organism. Obviously, this is even more important in the field of neuroscience where neural circuits perform computations dependent on their structure.

Currently, most analyses are two-dimensional. However, biological systems are 3-dimensional. Therefore, future analyses should be performed in three dimensions. Modern two-photon laser scanning microscopes and light-sheet microscopy systems should make it possible to image intact organs or whole animals in real time. Experiments using these image techniques generate huge data sets that require fast computers with large storage capacities to perform network analyses.

In the future, network analyses may benefit from employing more sophisticated methods to identify network links. This may include Granger causality test, which is a statistical hypothesis test for determining whether one time series is useful in forecasting another (Seth, 2010). Furthermore, both cross-correlation analysis and Granger causality detects linear relationships, opening up for further improvement for detection of non-linear connections such as transfer entropy (Vicente et al., 2011).

## Author contributions

Erik Smedler, Seth Malmersjö, and Per Uhlén designed the algorithm. Erik Smedler and Seth Malmersjö wrote the code. Erik Smedler and Seth Malmersjö analyzed the data. Erik Smedler, Seth Malmersjö, and Per Uhlén wrote the manuscript.

### Conflict of interest statement

The authors declare that the research was conducted in the absence of any commercial or financial relationships that could be construed as a potential conflict of interest.
