# Supplementary figures and images for: Network analysis of time-lapse microscopy recordings
Source: Front Neural Circuits. 2014 Sep 17;8:111. doi: 10.3389/fncir.2014.00111 (PMC4166320; doi:10.3389/fncir.2014.00111)

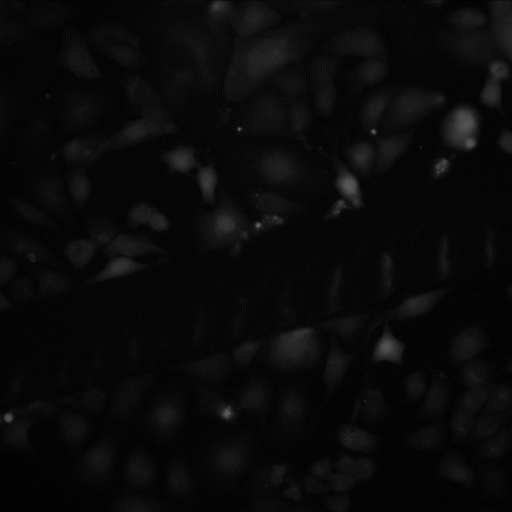

Supplement: Supplementary file 3 [file Presentation1.ZIP › mic2net/Example files/microscope_image.tif]

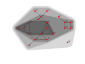

Supplement: Supplementary file 3 [file Presentation1.ZIP › mic2net/matlab_bgl/doc/html/core_numbers_example/core_numbers_example.png]

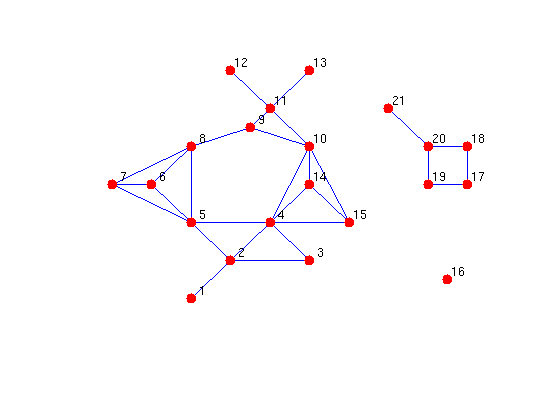

Supplement: Supplementary file 3 [file Presentation1.ZIP › mic2net/matlab_bgl/doc/html/core_numbers_example/core_numbers_example_01.png]

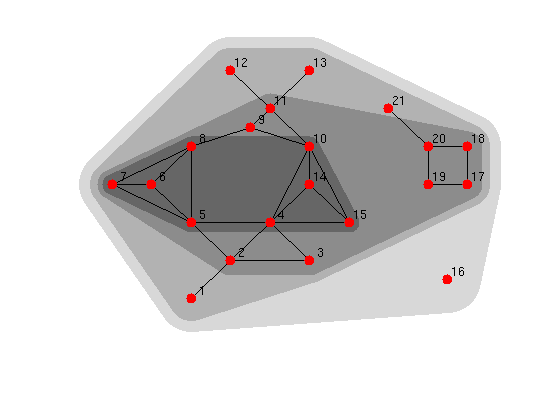

Supplement: Supplementary file 3 [file Presentation1.ZIP › mic2net/matlab_bgl/doc/html/core_numbers_example/core_numbers_example_02.png]

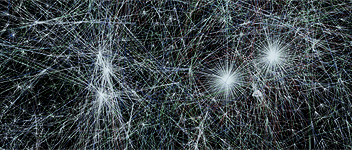

Supplement: Supplementary file 3 [file Presentation1.ZIP › mic2net/matlab_bgl/doc/html/images/matlab-bgl-header.png]

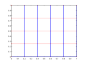

Supplement: Supplementary file 3 [file Presentation1.ZIP › mic2net/matlab_bgl/doc/html/new_in_3/new_in_3_0.png]

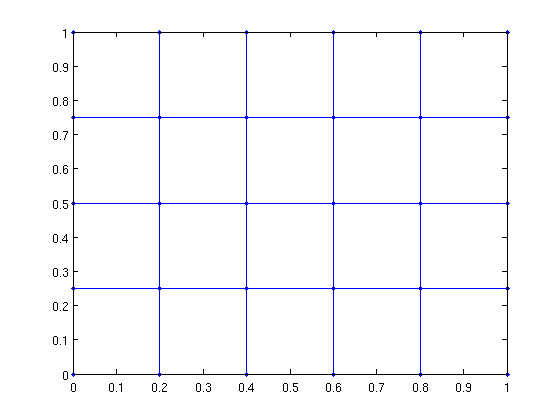

Supplement: Supplementary file 3 [file Presentation1.ZIP › mic2net/matlab_bgl/doc/html/new_in_3/new_in_3_0_01.png]

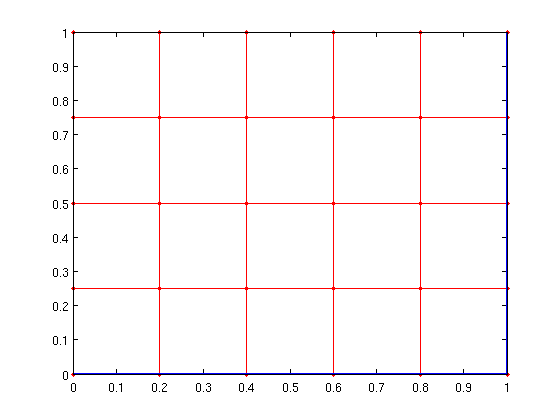

Supplement: Supplementary file 3 [file Presentation1.ZIP › mic2net/matlab_bgl/doc/html/new_in_3/new_in_3_0_02.png]

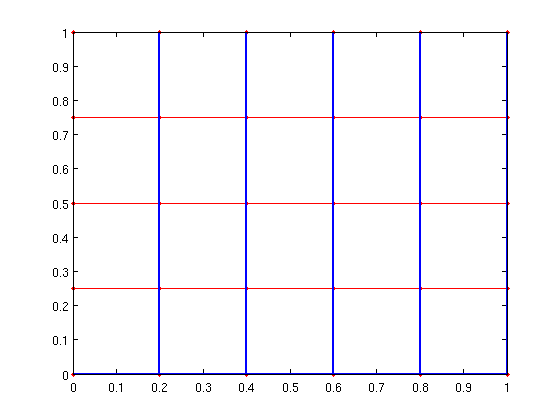

Supplement: Supplementary file 3 [file Presentation1.ZIP › mic2net/matlab_bgl/doc/html/new_in_3/new_in_3_0_03.png]

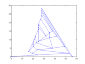

Supplement: Supplementary file 3 [file Presentation1.ZIP › mic2net/matlab_bgl/doc/html/new_in_4/new_in_4_0.png]

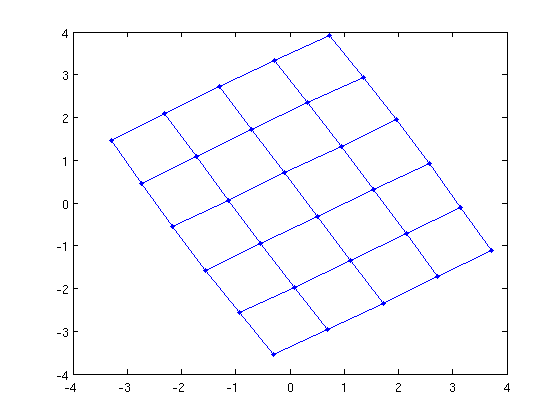

Supplement: Supplementary file 3 [file Presentation1.ZIP › mic2net/matlab_bgl/doc/html/new_in_4/new_in_4_0_01.png]

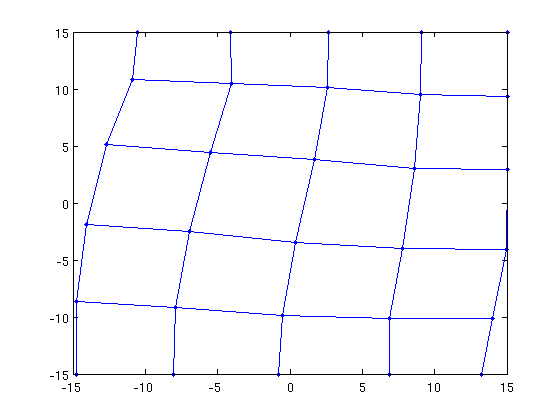

Supplement: Supplementary file 3 [file Presentation1.ZIP › mic2net/matlab_bgl/doc/html/new_in_4/new_in_4_0_02.png]

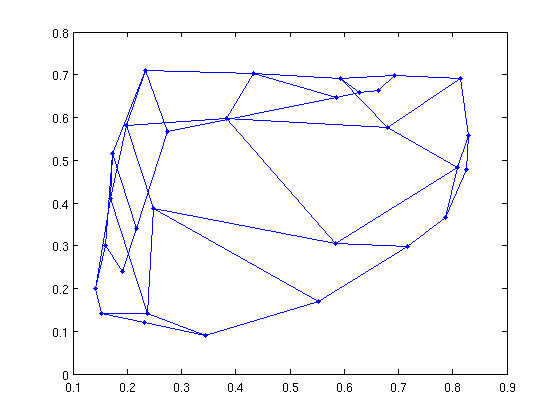

Supplement: Supplementary file 3 [file Presentation1.ZIP › mic2net/matlab_bgl/doc/html/new_in_4/new_in_4_0_03.png]

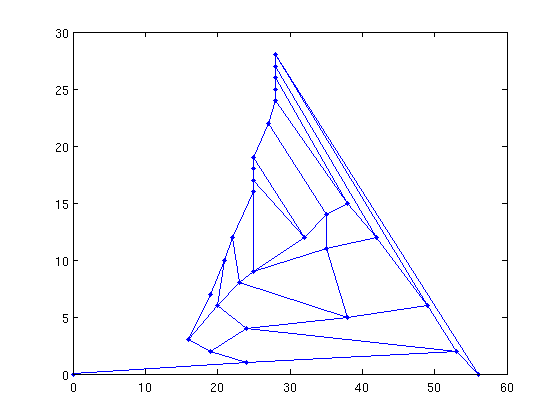

Supplement: Supplementary file 3 [file Presentation1.ZIP › mic2net/matlab_bgl/doc/html/new_in_4/new_in_4_0_04.png]

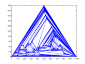

Supplement: Supplementary file 3 [file Presentation1.ZIP › mic2net/matlab_bgl/doc/html/planar_graphs/planar_graphs.png]

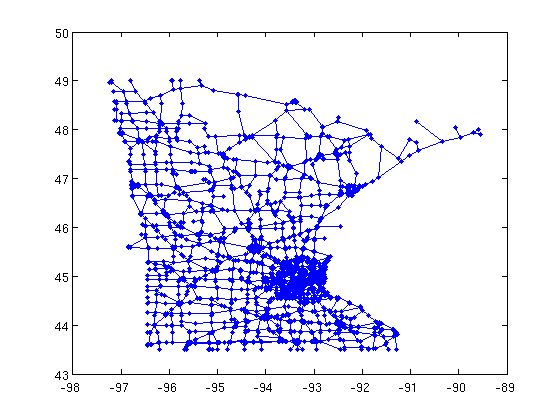

Supplement: Supplementary file 3 [file Presentation1.ZIP › mic2net/matlab_bgl/doc/html/planar_graphs/planar_graphs_01.png]

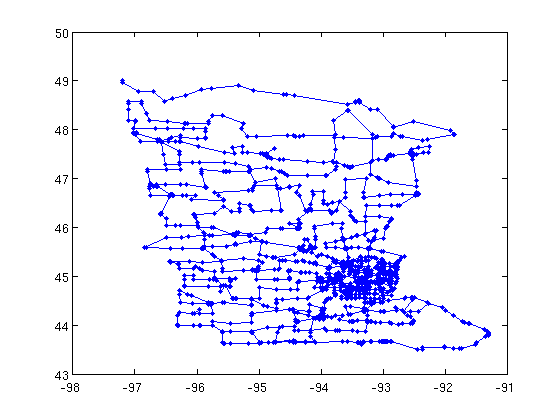

Supplement: Supplementary file 3 [file Presentation1.ZIP › mic2net/matlab_bgl/doc/html/planar_graphs/planar_graphs_02.png]

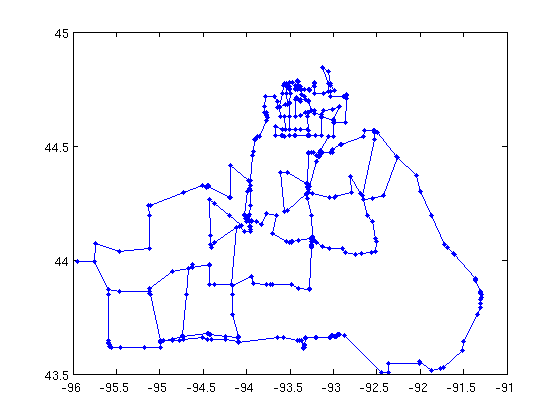

Supplement: Supplementary file 3 [file Presentation1.ZIP › mic2net/matlab_bgl/doc/html/planar_graphs/planar_graphs_03.png]

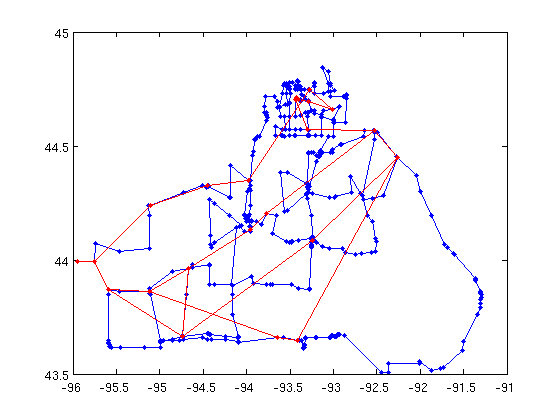

Supplement: Supplementary file 3 [file Presentation1.ZIP › mic2net/matlab_bgl/doc/html/planar_graphs/planar_graphs_04.png]

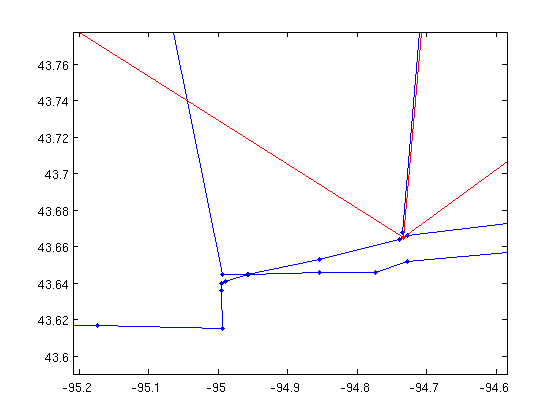

Supplement: Supplementary file 3 [file Presentation1.ZIP › mic2net/matlab_bgl/doc/html/planar_graphs/planar_graphs_05.png]

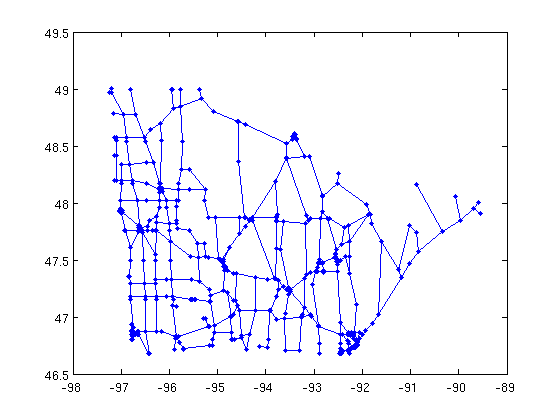

Supplement: Supplementary file 3 [file Presentation1.ZIP › mic2net/matlab_bgl/doc/html/planar_graphs/planar_graphs_06.png]

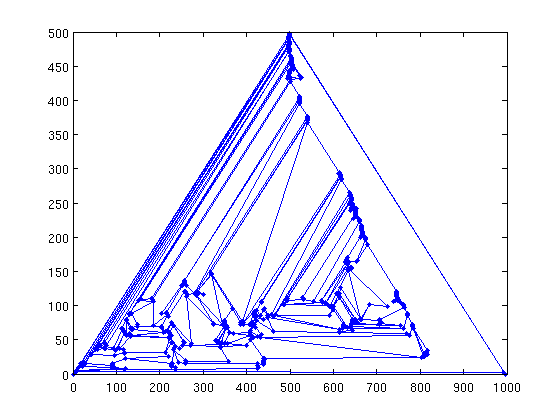

Supplement: Supplementary file 3 [file Presentation1.ZIP › mic2net/matlab_bgl/doc/html/planar_graphs/planar_graphs_07.png]

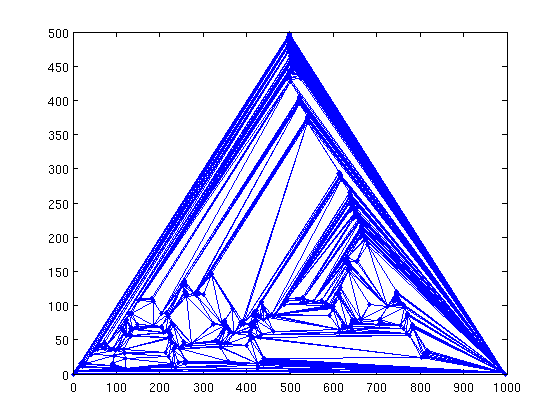

Supplement: Supplementary file 3 [file Presentation1.ZIP › mic2net/matlab_bgl/doc/html/planar_graphs/planar_graphs_08.png]

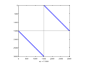

Supplement: Supplementary file 3 [file Presentation1.ZIP › mic2net/matlab_bgl/doc/html/red_black/red_black.png]

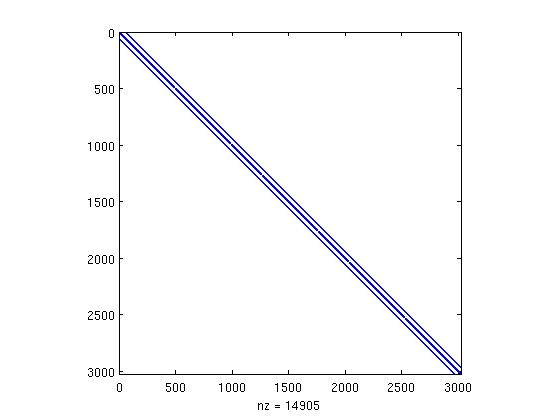

Supplement: Supplementary file 3 [file Presentation1.ZIP › mic2net/matlab_bgl/doc/html/red_black/red_black_01.png]

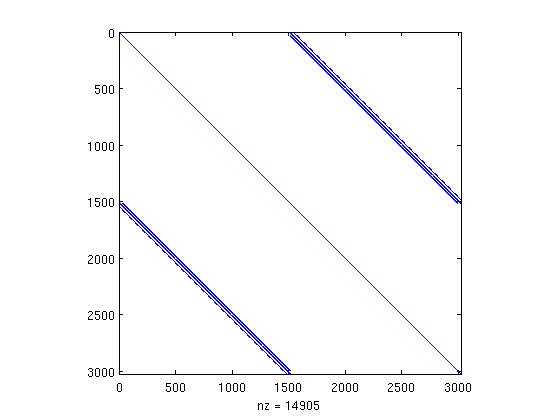

Supplement: Supplementary file 3 [file Presentation1.ZIP › mic2net/matlab_bgl/doc/html/red_black/red_black_02.png]

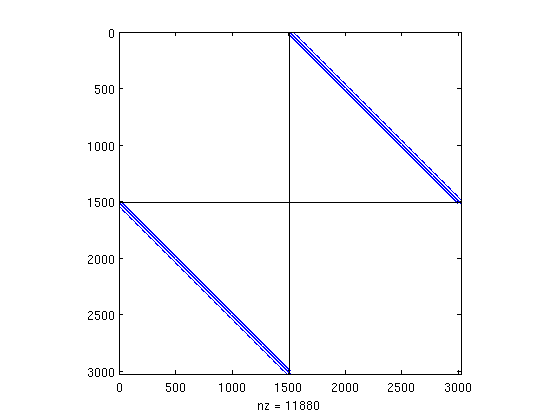

Supplement: Supplementary file 3 [file Presentation1.ZIP › mic2net/matlab_bgl/doc/html/red_black/red_black_03.png]

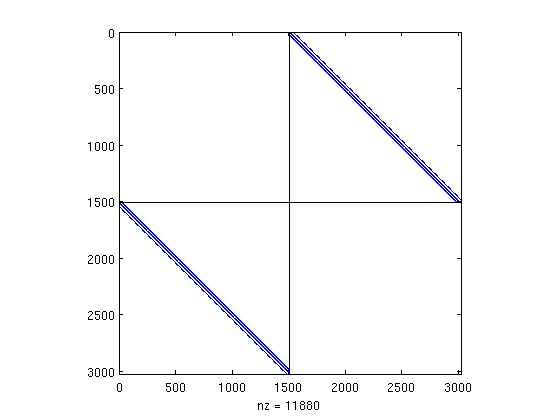

Supplement: Supplementary file 3 [file Presentation1.ZIP › mic2net/matlab_bgl/doc/html/red_black/red_black_04.png]
